# Data set on stability comparison of emulsions stabilized by cationic fluorosurfactant against conventional surfactants and high thermal performance of fluoropolymer foams

**DOI:** 10.1016/j.dib.2017.06.005

**Published:** 2017-06-06

**Authors:** Umair Azhar, Rimsha Yaqub, Bing Geng, Shuxiang Zhang

**Affiliations:** a Shandong Provincial Key Laboratory of Fluorine Chemistry and Chemical Materials, School of Chemistry and Chemical Engineering, University of Jinan, Jinan 250022, China; b Department of Chemistry, University of Gujrat, Gujrat, Pakistan

## Abstract

This data article includes emulsion stability comparison of cationic fluorosurfactant (CFS) against conventional surfactants. Span 80, Hypermer, Tween 80 and CTAB were used as conventional emulsifiers and only after 30 minutes bilayer phase separation observed in emulsions prepared by Tween 80 while CTAB failed to give fluoroemulsion, as compared to the CFS stabilized fluoro-HIPE which demonstrated superb stabilization of more than 72 h without phase separation. Thermal stability of Poly(hexafluorobutyl acrylate)-Divinyl benzene (PHFBA-DVB) was compared with porous polymer prepared by the same concentration of CFS 9 wt% by using trifluoroethyl methacrylate (TFEMA) as monomer phase. Results of PFP prepared with HFBA showed remarkable stability performance at more than 340.69 °C while porous polymer synthesized by TFEMA started to decompose even at 237.36 °C. The main findings based on the data presented here are reported in the paper “A cationicfluorosurfactant for fabrication of high-performance fluoropolymer foams with controllable morphology” (Azhar et al., 2017) [1].

**Specifications Table**

**Table:** 

Subject area	*Chemistry*
More specific subject area	*Organic chemistry*
Type of data	*Figure, Graph*
How data was acquired	*Digital camera(Cannon PowerShot SX210), Thermogravimetric analysis (TGA) was performed on Pyris Diamond TG/DTA (Perkin-Elmer Co.,USA),Contact angle instrument OCA dropshape analyzer (Data physics Co., Germany) for hydrophobicity test*
Data format	*Analyzed*
Experimental factors	*For thermogrevimetric analysis, the polymer was grinded to form a powder before analysis.*
Experimental features	*TGA was performed at heating rate of 10 °C/min and a scanning range of 30 to 550 °C in nitrogen atmosphere, Contact angles images were taken after 2 minutes of droplets contact with polymer*
Data source location	*Jinan, China*
Data accessibility	*Data is with this article*

**Value of the data**

• The data shows the importance of cationic fluorosurfactant in emulsion stability.
• This data may help to replace conventional surfactants which are unable to emulsify most of the fluoro-emulsion systems.
• It has been demonstrated in this data that the fluoropolymer shows high thermal stability up to 340° C.
• This data set will be beneficial for the researchers who want to work on high performance porous fluoropolymer foams.

## 1 Data

**Fig. 1:**
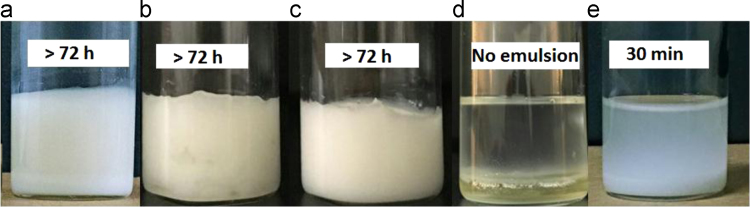
Stability comparison of fluoroHIPE systems stabilized by: (a) CFS, (b) Span 80, (c) Hypermer, (d) CTAB, and (e) Tween 80.

**Fig. 2:**
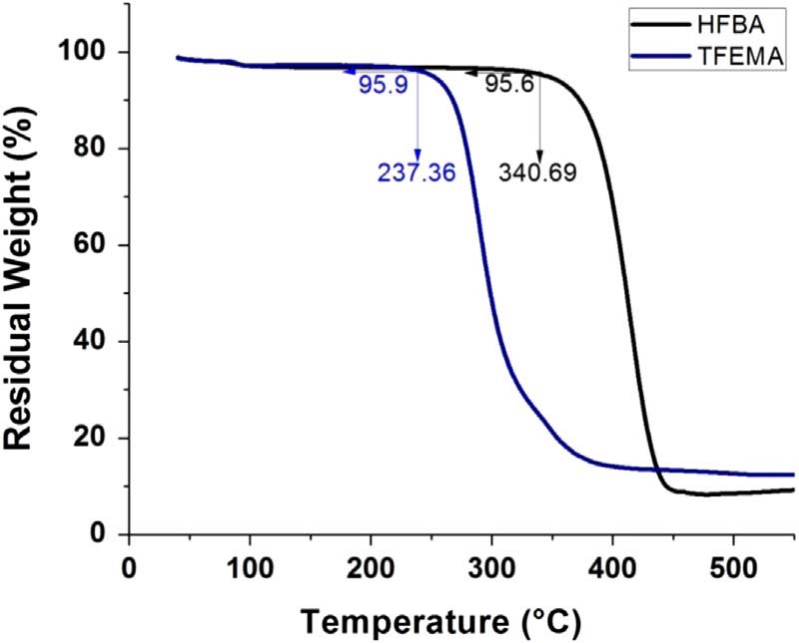
Effect of change in monomers on thermal stability.

**Fig. 3:**
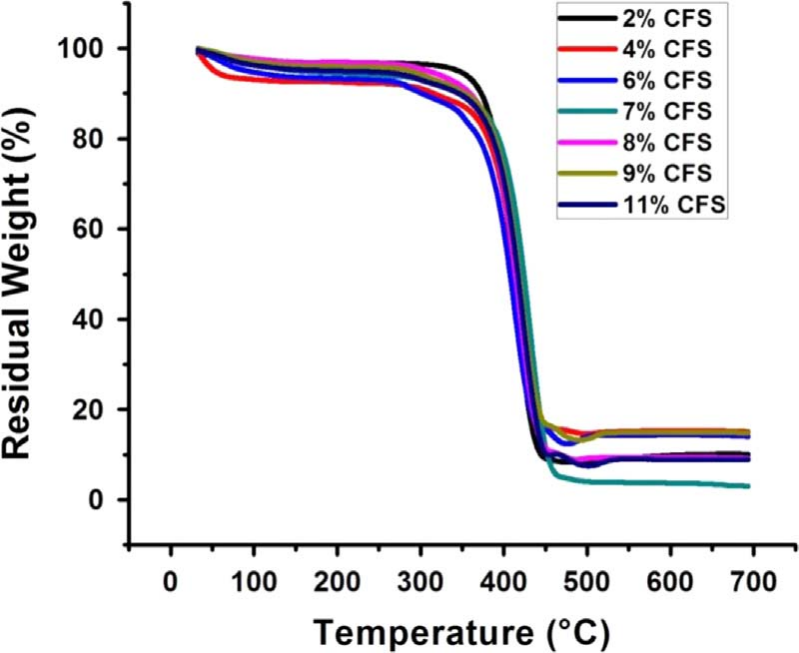
Effect of change in concentration of CFS on thermal stability.

**Fig. 4:**
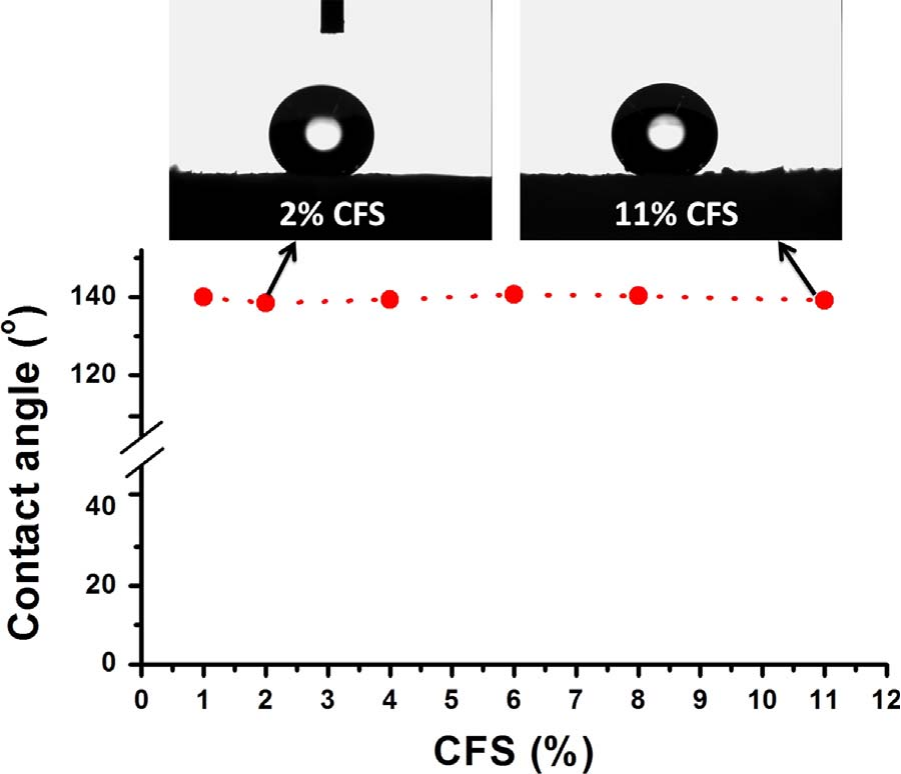
Effect of change in concentration of CFS on hydrophobicity.

There is an increasing interest in fluorine containing oils and fluorinated surfactants/block copolymers in the field of emulsion polymerization due to their magnificent advantages over hydrocarbons just like compatibility with numerous solvents, environmental stability, optical transparency, hydrophobicity and excellent chemical/oxidative stability [2], [3]. Fig. 1 describes the stability of fluoroemulsions prepared by different surfactants with same 9 wt% concentration in each vial. High performance of fluoropolymer foams and importance of fluorine contents in fluoropolymer are described in Fig. 2. The effect of cationic fluorosurfactant concentration on thermal stability and hydrophobicity of fluoropolymer foam is depicted in Figs. 3 and 4 respectively.

## 2 Experimental design, materials and methods

### 2.1 Emulsion stability

After preparation of high internal phase emulsions, these were taken in glass vials for digital photographs. As shown in Fig. 1, the emulsion prepared by cationic fluorosurfactant(CFS), Span 80 and Hypermer demonstrated good stability, while conventional cationic surfactant (CTAB) were unable emulsify the fluoro-emulsion. In Figs. 1a and b it can be seen that emulsions are viscous and stable even after 72 h, but these emulsions did not give regular interconnected polyHIPE morphology [1].

### 2.2 Thermal performance of fluoropolymer

Fluoropolymers were prepared by polymerization and drying of high internal phase emulsion and samples were grinded to make in powder form. Then, thermogrevimetric analysis were performed by using Pyris Diamond TG/DTA (Perkin-Elmer Co., USA) with heating rate of 10 °C/min and a scanning range of 30 to 550 °C in nitrogen atmosphere. Fig. 2 depicts the comparison between thermal stabilities of two fluoromonomers. PolyHIPE composed with HFBA(6 fluorine atoms) as monomer, showed high thermal stability than that of the polymer prepared with TFEMA (3 fluorine atoms) as monomer. Effect of CFS % on thermal stability of fluoropolymer is illustrated in Fig. 3. Thermal performances were then compared with the recent literature report [4], in which thermal stability of the emulsions prepared from hydrocarbons was discussed.

## 3 Hydrophobicity of fluoropolymer

Hydrophobicity of the solid materials is commonly specified by the contact angle measurement. If the contact angle θ≤90° the surface is said to be hydrophilic and if θ≥90° then the surface will be hydrophobic [5]. Here in, a contact angle instrument OCA drop shape analyzer (Data physics Co., Germany) was used to measure the hydrophobicity of porous fluoropolymer at room temperature. Images were captured after 2 minutes of the droplet stay on surface. Poly(HFBA-DVB) showed superb hydrophobicity with water contact angles ranged in 140° at almost all concentrations of CFS.

## References

[1] Azhar U. (2017). A cationic fluorosurfactant for fabrication of high-performance fluoropolymer foams with controllable morphology. Mater. Des..

[2] Mohamed A. (2016). Effect of surfactant headgroup on low-fluorine-content CO2-philic hybrid surfactants. J. Supercrit. Fluids.

[3] Fraker C.A., Mendez A.J., Stabler C.L. (2011). Complementary methods for the determination of dissolved oxygen content in perfluorocarbon emulsions and other solutions. J Phys. Chem. B.

[4] Pérez-García M.G. (2015). Porous monoliths synthesized via polymerization of styrene and divinyl benzene in nonaqueous deep-eutectic solvent-based HIPEs. RSC Adv..

[5] Zhang B. (2007). A novel approach for the preparation of organic-siloxane oligomers and the creation of hydrophobic surface. Appl. Surf. Sci..

