# Supplementary figures and images for: Data set on stability comparison of emulsions stabilized by cationic fluorosurfactant against conventional surfactants and high thermal performance of fluoropolymer foams
Source: Data Brief. 2017 Jun 6;13:396–400. doi: 10.1016/j.dib.2017.06.005 (PMC5480818; doi:10.1016/j.dib.2017.06.005)

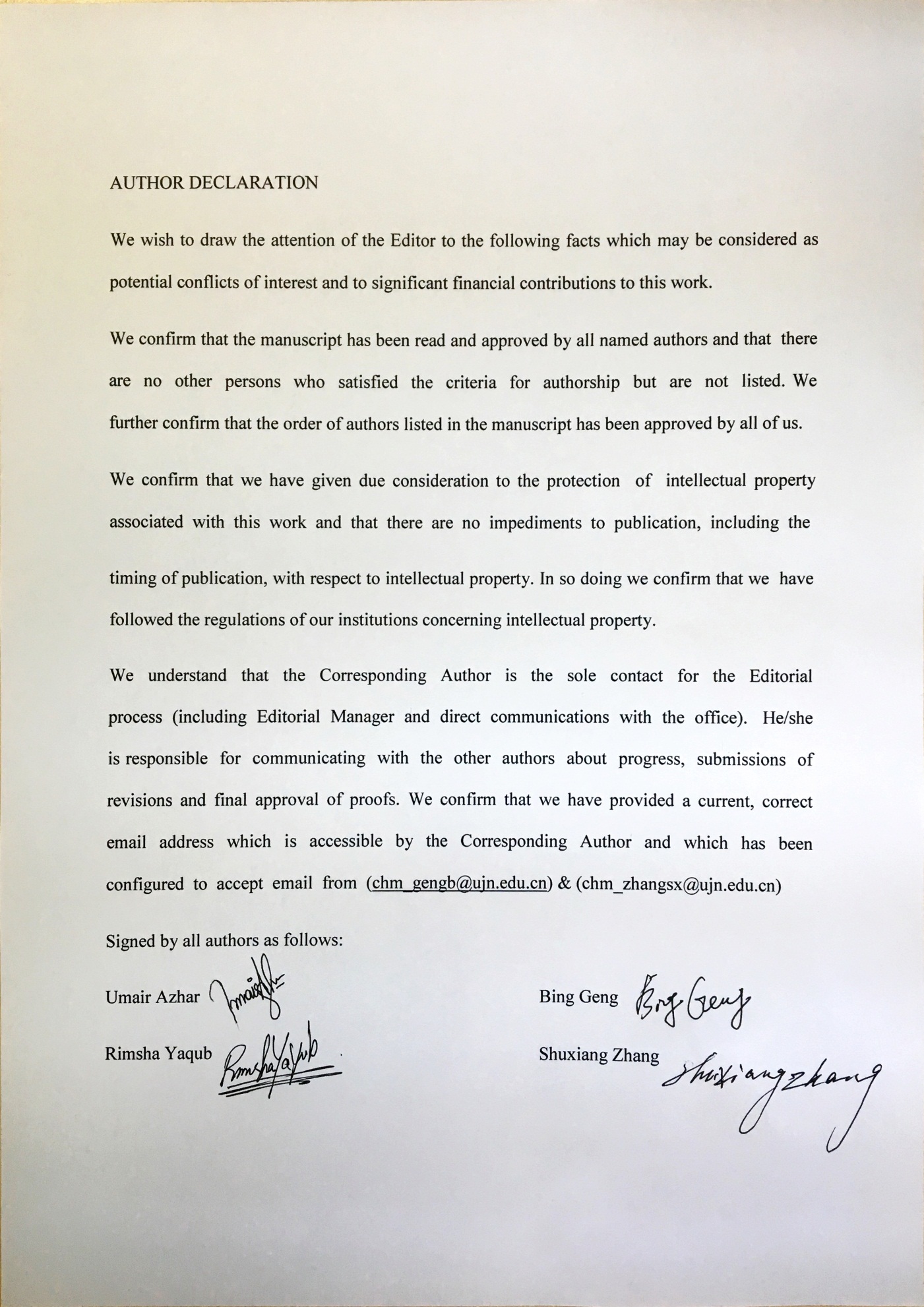

Supplement: Supplementary file 1 — Supplementary material [file mmc1.docx]
